# CAR T cell engineering approaches to minimise toxicities

**DOI:** 10.3389/ebm.2026.11021

**Published:** 2026-04-15

**Authors:** Elizabeth Hogben, Anna Schurich, Charlotte Graham

**Affiliations:** 1 Department of Haematology, King’s College London, London, United Kingdom; 2 Department of Haematology, King’s College Hospital NHS Foundation Trust, London, United Kingdom

**Keywords:** cytokine release syndrome (CRS), ICANS, non-ICANS neurotoxicity, safety, toxicity, CAR T cell

## Abstract

For the treatment of many forms of cancer, cell- and gene-based therapies are showing promise in both pre-clinical data and clinical trials. In particular, CAR T cell therapies, of which there are now 7 FDA-approved products, have shown ground-breaking results in haematological cancers such as multiple myeloma and B cell malignancies. Recent research is also attempting to develop effective CAR T cell therapies for solid tumours, with varying success. One of the key challenges faced by CAR T cell therapy is balancing strong cytotoxic activity for an effective treatment with preventing severe and potentially lethal toxicities, such as Cytokine Release Syndrome and Immune Effector Cell-Associated Neurotoxicity Syndrome. This mini review discusses some of the potential solutions that scientists have devised to overcome toxicities and improve existing CAR T cell therapies.

## Impact statement

We are delighted to submit this mini review titled “CAR T cell engineering approaches to minimise toxicities,” to be considered in the special issue focused on cell and gene therapy in the journal Experimental Biology and Medicine. CAR T cell therapy has transformed the treatment landscape of haematological malignancies, and is now being investigated for use in a range of autoimmune conditions and solid cancers. However, toxicity remains a major limitation to widespread use and limits the potential target antigens available. In this mini review we have highlighted some of the engineering strategies which have been developed to try and mitigate toxicity, and which we hope will lead to safer administration. Being able to safely manage more patients in the outpatient setting would increase capacity and ultimately widen access to these life-saving therapies.

## Introduction

Cell and gene therapies have made headlines in recent years through their potential demonstrated in pre-clinical data, with some breakthroughs translated into impressive clinical trial findings. A notable success story is CAR T cell therapy, which has become a lifeline for patients suffering from relapsed/refractory haematological malignancies, such as multiple myeloma (MM) and B cell acute lymphoblastic leukaemia (B-ALL). CAR T cell therapy involves isolating T cells from the patient’s blood and genetically modifying them by using a viral vector to express a chimeric antigen receptor (CAR). This CAR enables the T cell to precisely target a specific antigen expressed on cancer cells [1]. In the short history of CAR T cell research, CARs have had several generations of designs [2–4] with various modules included to boost co-stimulation of the T cell and increase its functionality. CAR T cells are designed to contain an antigen-binding domain attached to a transmembrane domain, which is most commonly linked to co-stimulatory domain(s) – such as CD28 or 41BB – and an activation domain (often CD3ζ) which enables downstream signalling and cytotoxic activity towards the target cell [5, 6].

Despite the rapid evolution of CAR T cell research, scientists still face common issues with toxicity and safety, such as Cytokine Release Syndrome (CRS) and Immune Effector Cell-Associated Neurotoxicity Syndrome (ICANS). CRS arises from a sharp increase in pro-inflammatory cytokines released into the bloodstream. Often, patients experience symptoms ranging from headaches and nausea to more severe complications, including hypotension and organ failure [7, 8]. Despite a lower incidence rate, ICANS is another serious toxicity associated with CAR T cell therapy where patients often experience symptoms of encephalopathy, headaches and tremor [9]. Non-ICANS neurotoxicities are a less frequent complication that can arise after CAR T cell infusion. Movement and neurocognitive toxicity (MNT) and cranial nerve palsy are associated with BCMA-targeting CAR T cell therapy, whereas complications such as myelopathy are described after CD19-targeting CAR T cell infusion [10]. In rare cases, haemophagocytic lymphohistiocytosis (HLH) is a hyperactive immune response which can be triggered by CAR T cell therapy and as such is defined as immune effector cell-associated HLH-like syndrome (IEC-HS) [11]. IEC-HS often manifests with symptoms such as coagulopathy, multiple classes of cytopenia and hepatic dysfunction [12, 13], but can be difficult to diagnose, with patients potentially displaying similarities to those with sepsis for example [14].

**FIGURE 1:**
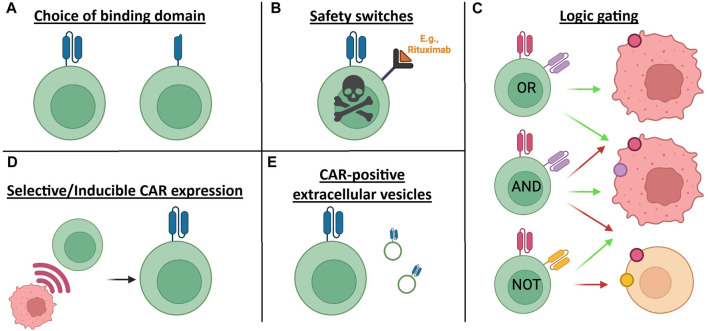
Summary diagram of the potential CAR T cell engineering approaches to tackle toxicities discussed in this mini review. **(A)** Choice of binding domain may influence the immunogenicity of a CAR T cell based on the inherent immunogenicity of the binder itself. For example, in this depiction, an scFv-based CAR is shown next to a nanobody-based CAR. **(B)** Safety switches can be incorporated within CAR design in the case of severe toxicities. **(C)** Logic gating is a more complex engineering approach to improving safety of CAR T cell therapies. In this figure the CAR colours match their respective target antigen, and the green arrows indicate a cytotoxic response to the target cell, whereas the red arrow indicates no cytotoxicity. **(D)** CARs can be selectively expressed in response to signals or other factors for example from the tumour microenvironment. **(E)** CAR T cells can secrete extracellular vesicles, expressing the CAR on their surface, which has generated discussion around the potential for their use as a cell-free alternative to CAR T cell therapy, with the possibility of reduced toxicities. Figure created in BioRender. Hogben, L. (2026) https://BioRender.com/bwfhcww.

The type and severity of toxicities faced by patients varies with their type of CAR T cell treatment. Typically, clinicians must closely monitor CAR T cell patients for signs of toxicities and treat the symptoms accordingly. In addition to the use of steroids and antipyretics to suppress inflammation, Tocilizumab is a monoclonal antibody which blocks the IL-6 receptor, and is often used to counteract CRS in the clinic [15, 16]. However, these methods fail to address the underlying cause of the toxicities and relies on prompt diagnosis of symptoms, rather than working as a preventative measure. This mini-review explores some of the novel approaches to prevent and minimise toxicities associated with CAR T cell therapy (Figure 1).

## Binder domains in CAR design

While each element of CAR T cell design may influence its efficacy, there has been much debate about the importance of the binding domain and its corresponding affinity and avidity effects. [17, 18]. Binding kinetics likely influence the secretion of pro-inflammatory cytokines and associated toxicities [19]. As the extracellular component, the properties of the binding domain are also hypothesised to impact the immunogenicity of the CAR T cell therapy and therefore possibly the likelihood of associated toxicities [20–22].

A key drawback for many of the approved CAR T cell therapies is that the antigen-binding domains are often based on a murine-derived scFv, FMC63 [23, 24], and as such are immunogenic due to sequence dissimilarities between murine and human VH and VL regions [25]. Indeed, there has been a case report of anaphylaxis caused by repeated CAR T infusions with murine derived sequences [22]. Humanisation strategies have been explored to reduce immunogenicity and improve safety in various studies [26, 27].

Notably, some studies have suggested nanobody-based CAR T cell designs show superior efficacy and safety over scFv-based designs [28], likely due to their increased stability and small size. Some studies have observed lower rates of CRS for nanobody-based CAR T cells [29], though this is disputed in other studies which suggest similar or increased rates of CRS [30]. One approved nanobody-based CAR T cell therapy, Ciltacabtagene autoleucel (Cilta-cel), has a binding domain comprising of two nanobody domains functioning similarly to an scFv [31]. This particular CAR T cell has shown breakthrough results rivalling its first-to-market competitor, Idecabtagene vicleucel (Ide-cel) which is based on a murine scFv targeting the tumour antigen, B cell maturation antigen (BCMA) [32]. Despite a recent finding suggesting Cilta-cel may be associated with a higher likelihood of grade 3 or higher CRS, there were no differences observed with the incidence of CRS in general or ICANS between Cilta-cel and Ide-cel [30]. Novel CAR T cell therapies with different binders are now challenging these market leaders for improved safety and efficacy. At the 2025 American Society of Haematology (ASH) meeting, Hart, *et al.* [33] discussed how the pharmacological profile of the D-Domain binder in Anitocabtagene autoleucel (Anito-cel) compares to a dual nanobody binder based on Cilta-cel, concluding a faster off-rate and “decreased cytokine production while maintaining similar cytotoxicity” as a result of the D-domain binder [33].

## Logic-gating

An ideal target for CAR T cell is one that is easily accessible and ideally exclusively, or highly differentially, expressed by the target cell, however this is not a common occurrence in biology. Of the seven FDA-approved CAR T cell therapies at the time of writing, five target CD19 and two target BCMA. Although it is the predominant target for these therapies, CD19 is a protein found on both healthy and malignant B cells and therefore an expected, undesirable side effect is B cell aplasia [34]. BCMA can be considered a favourable target as it is found on mature plasma cells, with its overexpression linked to multiple myeloma. Based on this differential expression, it could be suggested there is a lower risk of off-tumour on-target effects, though it is still found on haematopoietic stem cells to a lower degree. [35]. There have also been reports of BCMA expression within the central nervous system (CNS) and this may be associated with the development of a rare late neurological complication resembling Parkinson’s disease [36]. This is a similar problem with many drug products, in particular CAR T cell therapy, emphasising the need to improve specificity when targeting tumours.

**FIGURE 2:**
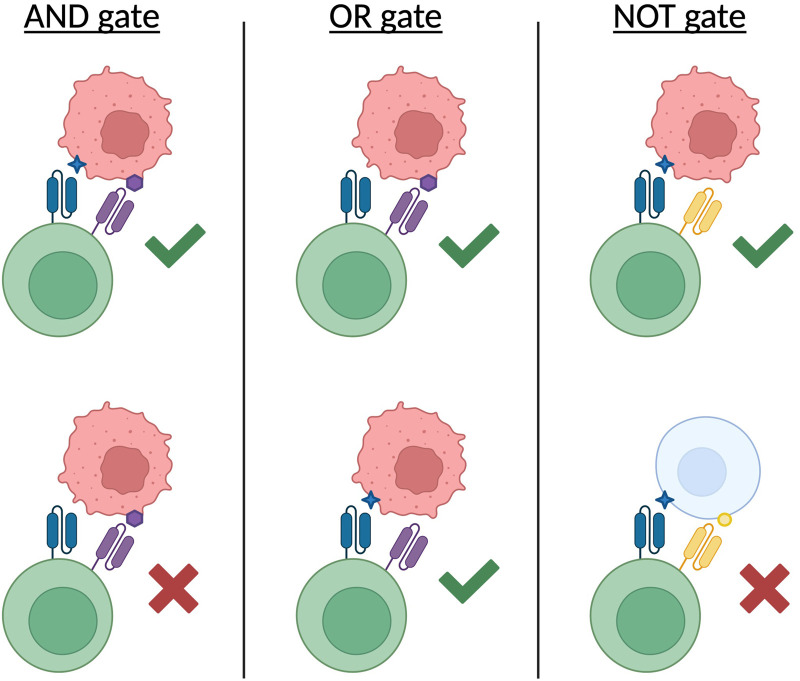
A graphic demonstrating activation conditions of three types of logic-gated CAR T cells. Green ticks indicate the combination of recognised antigens on the target cell results in CAR-mediated activation of the T cell (green cell). Red crosses convey no activation from the CAR(s) recognising the antigen(s). Figure created in BioRender. Hogben, L. (2026) https://BioRender.com/1s16uof.

As such, inspired by “Boolean logic” in computing, logic gating was engineered in CAR T cell therapies, incorporating “AND”, “OR” and “NOT” gates to improve targeting of tumour cells and reduce toxicities [37, 38]. Each gate operates under a different principle: “AND” gates require both target antigens to be present for CAR T cell activation and cytotoxicity; “OR” gates target two antigens but only one is required for activation; and “NOT” gates also target two antigens, though if both or only the inhibitory target are present, activation and cytotoxicity is prevented (Figure 2). “AND” and “NOT” gates are most suitable in reducing on-target off-tumour effects as their stringent conditional logic makes these toxicities less likely. This was exemplified in a pioneering study; Kloss, *et al.* [39] demonstrated that AND-gated CAR T cells, targeting CD19 and PMSA, elevated cytotoxicity towards cells expressing both antigens, through the co-stimulation of CD28 and 41BB on distinct CAR modules. They also showed that without the co-stimulation from both receptors, and therefore recognition of both antigens, tumour cells were relatively unaffected, suggesting this approach could avoid off-tumour on-target effects where antigens are expressed by healthy tissues as well [39]. In principle, the targeting of two tumour antigens may overcome tumour resistance mechanisms such as antigen escape due to the low likelihood of splicing and mutation events for both of these antigens, however it does increase the selection pressure in the event of any tumour clones lacking these target antigens [40]. Additionally, in the case of AND-gated CAR T cells, it could decrease efficacy where some tumour clones may lack one of the antigens required for CAR T cell activation. Overall, this study provides proof-of-concept for the potential of logic-gated CARs, with recent work from other groups publishing similar *in vitro* findings for AND-gated CARs relevant to multiple myeloma [41] and other haematological or solid tumour targets [42].

## Selective CAR expression

Like the concept of logic-gating, selective expression of CARs under specific conditions is another avenue being researched to prevent toxicities. Rather than relying on certain combinations of antigens present on tumour cells, specific promoters can induce CAR expression under specific conditions for example as found in the tumour microenvironment (TME) [43, 44], or in response to drugs administered [45, 46].

One of the challenges faced by CAR T cell therapy in treating solid tumours in particular is balancing the need for a highly effective CAR T cell, which can penetrate and clear tumour burden, whilst also preventing severe toxicities such as CRS and ICANS. The TME of solid tumours is known to be immunosuppressive with a dense extracellular matrix (ECM), which can cause reduced oxygen supply to the core of the tumour. Kosti, *et al.* [44] utilised this tumour property to develop a HypoxiCAR which would stringently express the CAR under hypoxic conditions, as may be found in the TME. ErbB-targeting CAR T cell therapies have previously been deemed unsuitable for intravenous infusion based on severe toxicities, including the death of a patient [47], associated with the broad expression of the target antigen. In this study they demonstrated that a pan-ErbB-targeting CAR could be expressed under hypoxic, but not normoxic, conditions using a hypoxia-responsive promoter. *In vitro* and *in vivo* data showed reduced cytokine secretion as well as off-tumour infiltration into healthy organs, suggesting potentially improved safety [44]. Another group has also demonstrated hypoxia-sensing small molecules can be used to permit CAR expression exclusively in response to factors in the TME [43]. Similarly, other groups have developed expression systems combining logic-gating whereby CARs are only expressed upon recognition of a combination of antigens present on the tumour cells through the use of synthetic Notch (SynNotch) mechanisms that induce expression, resulting in reduced toxicity from off-tumour on-target effects [48, 49]. These mechanisms are based on the highly conserved Notch signalling pathway, whereby a SynNotch receptor contains the extracellular binding domain(s), the Notch juxtamembrane and transmembrane domains, and intracellular domains with orthogonal transcription factors (TFs). If the mechanism is designed so that the CAR gene is under the influence of these orthogonal TFs, when the SynNotch receptor binds its target antigen, the mechanism becomes activated, allowing the TF to trigger CAR expression on the surface of the T cell. This combinatorial activation mechanism means that the CAR is only expressed when the antigen recognised by the SynNotch receptor is present, and therefore subsequent cytotoxicity of the CAR T cell can only occur when both this antigen, and the CAR’s target antigen is present [50].

## Safety switches

Just as CAR expression can be induced under specific conditions, the importance of being able to “turn off” CAR expression has also been addressed. In response to safety concerns, some scientists have incorporated safety switch mechanisms in their CAR designs. These mechanisms often rely on the addition of a drug or small molecule to trigger apoptosis in the CAR T cells in the event of severe toxicities.

Early examples of these safety switches include the HSV-tk (Herpes Simplex Virus Thymidine kinase) system which conveys sensitivity to Ganciclovir to downregulate graft versus host disease (GvHD) in patients [51, 52], and the inducible caspase-9 (iCas9) system which triggers apoptosis of CAR T cells upon addition of Rimiducid [53]. More recently, the RQR8 safety switch has also been developed, providing an option to cause selective apoptosis of the CAR T cells containing this gene upon addition of Rituximab [54]. In addition, other CAR T cell designs have included tEGFR as a way to both identify transduced T cells during manufacture as well as potentially acting as a safety switch upon administration of Cetuximab, though this method can be ineffective at depleting the CAR T cells [55].

Whilst safety switches have been tested in clinical trials [56, 57] with mixed outcomes in neutralising toxicities, it is a similar approach to using Tocilizumab in that clinicians must administer the relevant drug reactively to symptoms of toxicities. Though this system does benefit from more direct elimination of the CAR T cell population to prevent toxicities, rather than alleviating the symptoms for example by blocking cytokines, it also abrogates the desired anti-tumour effects of the therapy and therefore should be as a last resort to minimising toxicities.

## CAR-positive extracellular vesicles

Recent studies have indicated that CAR-positive extracellular vesicles (CAR + EVs) are an important factor in the dynamics of CAR T cell activity. Some have suggested that levels of CAR + EVs can act as a predictor of ICANS, but not CRS, and therefore a potential biomarker for this toxicity [58]. However, other researchers have sought to pose CAR + EVs as a potential “cell-free” CAR T cell therapy, suggesting that CAR + EVs may also convey cytotoxicity towards target cells and may better penetrate the TME, whilst offering a safer, less immunogenic alternative to standard CAR T cell therapy [59, 60]. While this is a relatively new area of research within the CAR T cell field, it is an exciting development that could offer an avenue for off-the-shelf therapy with reduced safety concerns [61].

## Conclusion

Since its conception, CAR T cell therapy has rapidly evolved but has faced significant barriers with safety and toxicities requiring scientists to find solutions. As evidenced in this review, multiple avenues have been explored to reduce toxicities associated with CAR T cell therapy, though it is crucial to note that these are based on experimental data from pre-clinical studies, and do not take into account other important factors that would become relevant later in the clinical development. Pre-emptive solutions, such as logic-gating and selective expression systems, show promise in reducing the likelihood of CRS and ICANS in patients, though developments in alternative approaches to CAR-based therapies such as CAR + EVs are a compelling proposal to circumvent safety issues. Complex engineering approaches may introduce higher costs and greater manufacturing complexity, and potential concerns from regulatory boards. Through more clinically advanced studies and with larger scale production, the combination of safety, efficacy and practicality of manufacturing will determine CAR T cell success.
